# Endoplasmic Reticulum Autophagy Enhancement Alleviates Synaptic Plasticity Dysfunction and Ameliorates Cognitive Impairment in SAE Mice by Relieving STING‐Associated Inhibition of PKM2 Activity

**DOI:** 10.1002/cns.71233

**Published:** 2026-10-29

**Authors:** Xiaofeng Guo, Daqing Yin, Zhonghua Zhang, Yuanyuan Zhao, Yali Zhang, Ying Jiang, Zhanying Zhao, Tongwen Sun, Feihu Zhou, Xijing Zhang

**Affiliations:** ^1^ Department of Critical Care Medicine The First Medical Center of Chinese PLA General Hospital Beijing China; ^2^ Department of Critical Care Medicine The 988th Hospital of the PLA Joint Logistics Support Force Zhengzhou Henan China; ^3^ Department of Critical Care Medicine Xijing Hospital, Air Force Medical University Xi'an Shaanxi China; ^4^ Department of Critical Care Medicine The First Affiliated Hospital of Zhengzhou University Zhengzhou Henan China

**Keywords:** micro‐ER‐phagy, pyruvate kinase M2 (PKM2), sepsis‐associated encephalopathy, stimulator of interferon genes (STING), synaptic plasticity

## Abstract

**Background:**

The pathogenesis of cognitive impairment in patients with sepsis‐associated encephalopathy (SAE) remains unclear. This study investigated whether enhancing micro‐endoplasmic reticulum autophagy (micro‐ER‐phagy) could alleviate synaptic dysfunction and improve cognitive impairment in SAE mice models by relieving stimulator of interferon gene (STING)‐associated inhibition of pyruvate kinase M2 (PKM2) activity.

**Methods:**

Male wild‐type and neuron‐specific PKM2 conditional knockout mice (*PKM2* ^fl/fl^; hSyn‐Cre^+^) were used. Cognitive function in SAE mice models was assessed using the Y‐maze and contextual fear conditioning tests. Neuronal plasticity was examined using transmission electron microscopy and Golgi staining, whereas immunofluorescence staining and Western blotting were employed to determine the temporal and spatial expression patterns of related proteins.

**Results:**

Septic mice developed cognitive impairment. Hippocampal tissues of SAE mice exhibited synaptic plasticity dysfunction and glycolysis enhancement. Meanwhile, the hippocampus showed increased PKM2 expression and an altered ratio of PKM2 tetramers and dimers. In addition, phosphorylated STING significantly increased in concentration in hippocampal neurones and its knockdown improved cognitive function. However, this cognitive rescue failed in PKM2 conditional knockout mice treated with SAE. Finally, Sec62‐mediated micro‐ER‐phagy promoted lysosomal degradation of STING, which relieved STING‐mediated inhibition of PKM2 and improved synaptic plasticity and cognitive dysfunction in SAE mice.

**Conclusions:**

Elevated micro‐ER‐phagy may relieve the STING‐associated inhibition of PKM2 activity, improve neuronal glycolysis and synaptic plasticity, and alleviate cognitive dysfunction in SAE mice.

## Graphical abstract

In sepsis‐associated encephalopathy (SAE), enhanced micro‐ER‐phagy mediated by Sec62 facilitates the delivery of STING to lysosomes for degradation in hippocampal neurons. This process relieves STING‐mediated inhibition of PKM2 activity, which further modulates cellular energy metabolism indicators including the ATP/ADP and NAD^+^/NADH ratios. Consequently, neuronal synaptic plasticity is preserved, ultimately ameliorating cognitive impairment in SAE mice.

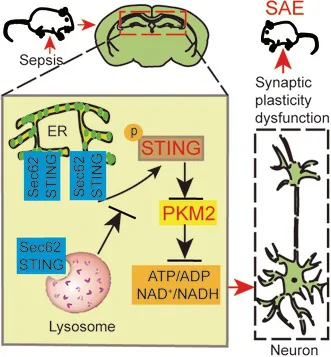

## 1 Introduction

Sepsis‐associated encephalopathy (SAE) is characterized by diffuse cerebral dysfunction induced by sepsis‐mediated systemic inflammation independent of central nervous system infection [1]. Approximately 20% of surviving patients with SAE experience sustained cognitive impairment [2, 3]. The pathogenesis of SAE may be associated with neuroinflammatory responses, synaptic dysfunction, and cell death [4, 5]; however, the precise mechanism remains unclear.

Synaptic plasticity is highly sensitive to metabolic insufficiency [6, 7, 8, 9, 10, 11, 12, 13, 14]. Recent studies have shown that the glycolytic enzyme pyruvate kinase M2 (PKM2) drives neuronal energy metabolism under stress [15, 16, 17, 18]. It oscillates between an active tetramer and a low‐activity dimer [19]. However, the exact mechanisms governing PKM2 expression in the hippocampal tissues of SAE mice remain unclear.

The stimulator of interferon gene (STING) regulates cellular energy metabolism and inflammatory responses [20, 21]. In tumor cells, STING inhibits glycolysis‐mediated ATP production, which, in turn, affects tumor cells [20, 22]. Accumulating evidence has demonstrated that PKM2 is essential for glycolytic metabolism [15, 16, 18]. In PKM2 conditional knockout mice with ischaemic stroke, increased neuronal death was observed; however, neuronal damage was significantly reduced after pyruvate supplementation [18, 23]. Therefore, we speculated that PKM2 acts as a critical downstream molecule of STING in the regulation of glycolysis. Our previous studies have demonstrated that STING downregulation attenuates neuronal damage in the hippocampus and rescues cognitive impairment in SAE mice [24]. However, further investigations are needed to determine whether STING regulates PKM2 during synaptic plasticity‐related energy metabolism in SAE mice.

Micro‐ER‐phagy is a cellular maintenance process in which the lysosomal membrane invaginates to directly engulf damaged ER components, effectively clearing waste and maintaining cellular homeostasis. While SEC62 is known to play a pivotal role in micro‐ER‐phagy [25, 26] and both SEC62 and STING share identical light chain 3 (LC3)‐interacting region (LIR) domains [26, 27, 28], it remains unclear whether micro‐ER‐phagy regulates STING during the progression of SAE.

In this study, we demonstrated that SAE mice had disrupted hippocampal glycolysis, impaired synaptic plasticity, and obvious cognitive dysfunction. Enhancing PKM2 activity improves glycolytic and synaptic deficits and ameliorates cognitive impairment in SAE mice. In addition, STING knockdown elevated PKM2 activity and ameliorated cognitive dysfunction in SAE mice. However, these protective effects were abrogated in the PKM2 cKO SAE mice. Enhancing micro‐ER‐phagy alleviated cognitive dysfunction in SAE mice by relieving STING‐mediated inhibition of PKM2 activity.

## 2 Materials and Methods

### 2.1 Animals and Euthanasia Protocol for Mice

Male wild‐type C57BL/6J mice (6‐ to 8‐week‐old) were purchased from Beijing Vital River Laboratory Animal Technology Co. Ltd. (Beijing, China). Male PKM2^fl/fl^; hSyn‐Cre^+^ (PKM2 cKO) and PKM2^fl/fl^; hSyn‐Cre^−^ (control) mice were obtained from the Air Force Medical University (Shaanxi, China).

In addition, experimental mice were euthanised in strict compliance with the Guide for the Care and Use of Laboratory Animals published by the National Institute of Health. The mice were euthanised by intraperitoneal injection of an overdose of pentobarbital sodium. The procedure was verified by confirming the cessation of both cardiac and respiratory functions before commencing tissue collection.

### 2.2 Cecal Ligation and Puncture and Grouping

A sepsis model was established via cecal ligation and puncture (CLP) surgery. Animals were anesthetized using isoflurane. After disinfection, a midline incision (approximately 1.0 cm in length) was made along the ventral midline of the lower and middle abdomen, followed by a sequential incision of the skin and peritoneum. Ligation was performed at the midpoint between the ileocecal valve and cecal apex. The needle (22‐G) was then used to puncture the halfway point between the ligation site and apex. A small volume of intestinal content (approximately the size of a rice grain) was gently squeezed out. Next, the peritoneum and skin were sutured layer‐by‐layer, and the incision was disinfected. Postoperative analgesia was administered with 0.1 mL of 2% lidocaine. The mice in the sham group underwent laparotomy without CLP.

As shown in Figure 1 and Figure 3, the mice were assigned to the Sham and CLP groups. As shown in Figure 2, the mice were randomly assigned to four groups: Sham + DMSO, Sham + DASA‐58, CLP + DMSO, and CLP + DASA‐58. The mice were randomly categorized into four groups: Sham + scRNA, Sham + STING‐shRNA, CLP + scRNA, and CLP + STING‐shRNA (Figure 4). As shown in Figure 5, the mice were randomly divided into four groups: Control + Sham + STING‐shRNA, PKM2 cKO + Sham + STING‐shRNA, Control + CLP + STING‐shRNA, and PKM2 cKO + CLP + STING‐shRNA. As shown in Figure 6, the mice were randomly assigned to four groups: Sham + AAV‐hSyn, Sham + AAV‐hSyn‐Sec62, CLP + AAV‐hSyn, and CLP + AAV‐hSyn‐Sec62.

**FIGURE 1:**
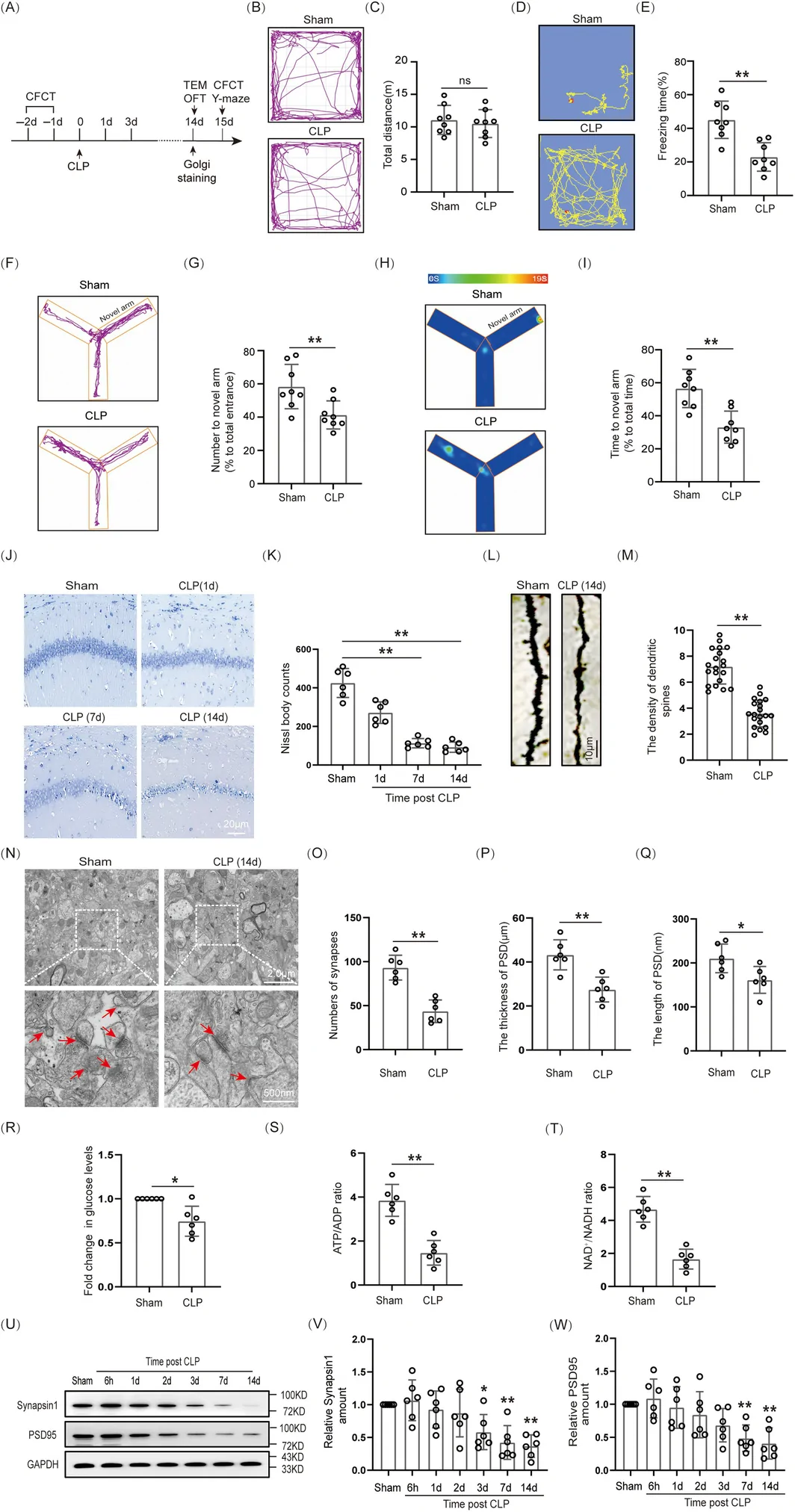
SAE alters glycolysis, synaptic plasticity and behavioral impairment. (A) Time diagram. (B) OFT trajectory diagram, (C) Total distance statistical results. (D) CFCT trajectory diagram, (E) Freezing time statistical results. (F) Y‐maze test trajectory diagram, (G) Number to novel arm statistical results. (H) Y‐maze test heatmap, (I) Time to novel arm statistical results. Statistical analysis: Student's *t*‐test. (J) Nissl staining, (K) Nissl bodies count statistical results (*F* = 57.54, *p* < 0.0001), scale bar 20 μm. Statistical analysis: One‐way ANOVA with Dunnett's multiple comparisons test. (L) Golgi staining, (M) Dendritic spine density statistical results, scale bar 10 μm. Statistical analysis: Nested test. (N) TEM images of synapses, (O–Q) synaptic plasticity statistical results, scale bar 2.0 μm and 500 nm. (R–T) Glucose concentration, ATP/ADP ratio, and NAD^+^/NADH ratio statistical results. Statistical analysis: Student's *t*‐test. (U) WB, (V, W) statistical results of synapsin 1 (*F* = 7.222, *p* < 0.0001) and PSD95 (*F* = 6.169, *p* = 0.0002). GAPDH was used as internal control. Statistical analysis: One‐way ANOVA with Dunnett's multiple comparisons test. ns: No significant, *p* > 0.05, *p < 0.05, **p < 0.01 versus sham.

**FIGURE 2:**
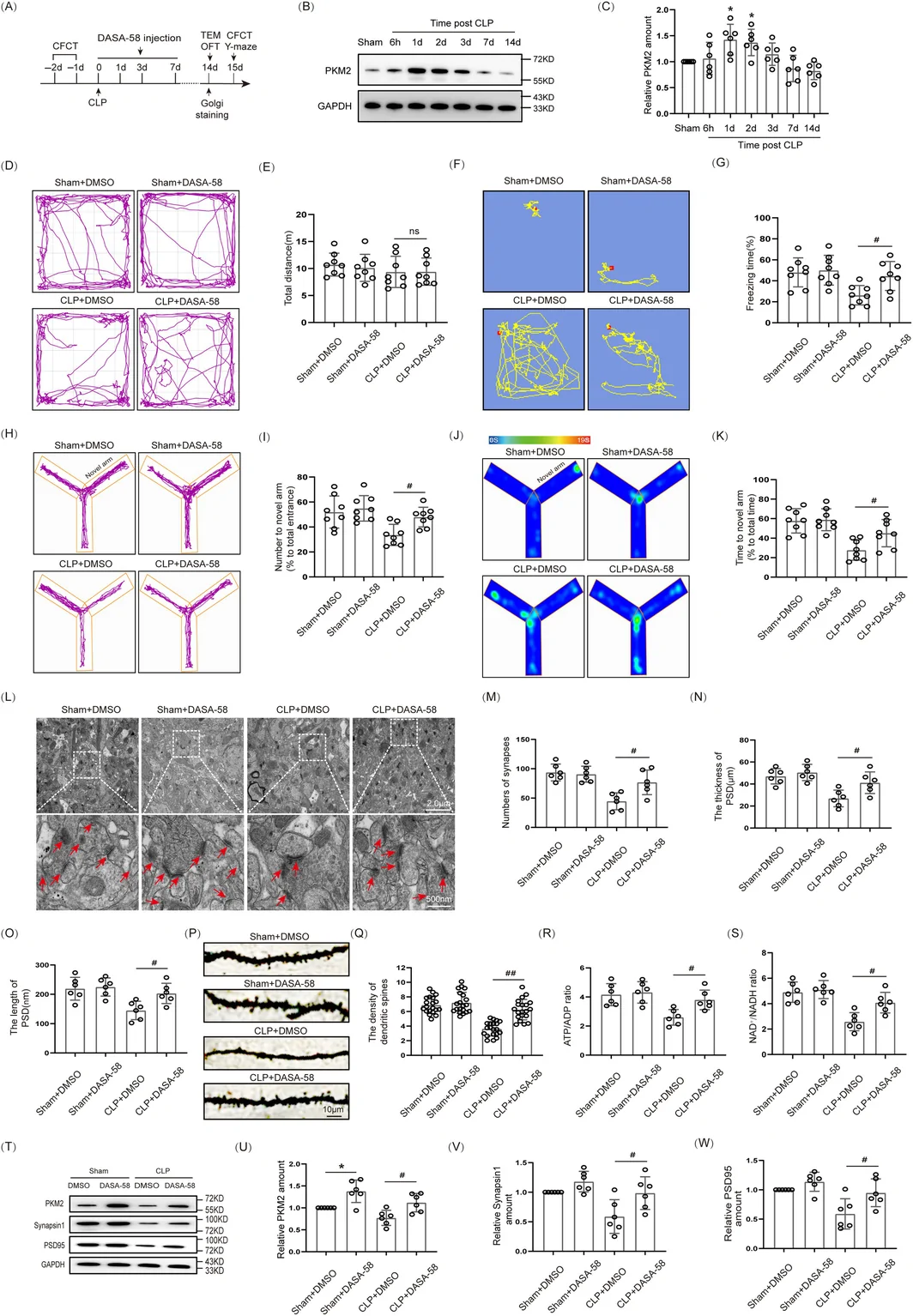
Enhancing PKM2 activity improves glycolysis, synaptic plasticity and behavioral impairment in SAE mice. (A) Time diagram. (B, T) WB, (C) statistical analysis of PKM2 (*F* = 5.846, *p* = 0.0003). GAPDH was used as internal control. Statistical analysis: One‐way ANOVA with Dunnett's multiple comparisons test. *p < 0.05 versus sham. (D) OFT trajectory diagram, (E) total distance statistical results (*F* = 0.1405, *p* = 0.7106). (F) CFCT trajectory diagram, (G) Freezing time statistical results (*F* = 4.981, *p* = 0.0338). (H) Y‐maze test trajectory diagram, (I) Number to novel arm statistical results (*F* = 5.930, *p* = 0.0215). (J) Y‐maze test heatmap, (K) Time to novel arm statistical results (*F* = 7.709, *p* = 0.0097). (L) TEM images of synapses, (M–O) Synaptic plasticity statistical results (*F* = 7.207, *p* = 0.0143; *F* = 7.290, *p* = 0.0138; *F* = 5.250, *p* = 0.0329), scale bar 2.0 μm and 500 nm. Statistical analysis: Two‐way ANOVA with Sidak's multiple comparisons test. (P) Golgi staining, (Q) Dendritic spine density statistical results (*F* = 33.63, *p <* 0.0001), scale bar 10 μm. Statistical analysis: Nested one‐way ANOVA with Dunnett's multiple comparisons test. (R, S, U–W) Statistical results of ATP/ADP ratio (*F* = 5.926, *p* = 0.0244) and NAD^+^/NADH ratio (*F* = 7.501, *p* = 0.0127), PKM2 (*F* = 22.65, *p* = 0.0001), synapsin 1 (*F* = 10.60, *p* = 0.0040), PSD95 (*F* = 9.830, *p* = 0.0052). GAPDH was used as internal control. Statistical analysis: Two‐way ANOVA with Sidak's multiple comparisons test. *p < 0.05 versus Sham + DMSO; ns: No significant, *p* > 0.05, ^#^ *p* < 0.05, ^##^ *p* < 0.01 versus CLP + DMSO.

### 2.3 Stereotaxic Injection

Three weeks before CLP, 300 nL of the viral vector (STING‐shRNA, scRNA, hIBA1a‐scRNA, hIBA1a‐STING‐shRNA, AAV‐hSyn‐Sec62, or AAV‐hSyn) was microinjected into the murine hippocampal CA1 region. rAAV2/9‐U6‐shRNA(STING)‐CMV‐mCherry‐SV40 polyA (STING‐shRNA) and rAAV2/9‐U6‐shRNA(scrambled)‐CMV‐mCherry‐SV40 polyA (scRNA) have been validated in our previous studies [24]. The shRNA target sequence was GCAACATTCGATTCCGAGATAT. The rAAV2/5‐hIBA1a‐EGFP‐5'miR‐30a‐shRNA(STING)‐3'miR‐30a‐miR‐124 T‐WPRE (hIBA1a‐STING‐shRNA) and rAAV2/5‐hIBA1a‐EGFP‐5'miR‐30a‐shRNA(scramble)‐3'miR‐30a‐miR‐124 T‐WPRE (hIBA1a‐scRNA) effectively downregulate STING expression in microglia. rAAV2/9‐hSyn‐Sec62‐P2A‐mCherry‐WPRE‐hGH polyA (AAV‐hSyn‐Sec62), rAAV2/9‐hSyn‐mCherry‐WPRE‐hGH polyA (AAV‐hSyn), LV‐CMV‐Sec62‐2a‐mCherry‐PGK‐Puro‐WPRE (LV‐Sec62) and LV‐CMV‐mCherry‐PGK‐Puro‐WPRE (LV‐Control) were used. Figure S1A shows the full‐length coding sequence (CDS) of mouse Sec62, covering nucleotides 473–1669 (CCDS17286.1). Previous studies have consistently validated that the hSyn‐Sec62 construct effectively activates ER‐phagy in neurons [29]. All the above rAAV vectors used in our experiments were purchased from Brain VTA Co. Ltd. (Wuhan, China). Surgery was guided by the following stereotaxic coordinates: AP: −2.0 mm, ML: ±1.60 mm, and DV: −1.6 mm. DASA‐58 (a PKM2 activator) was administered to mice starting on the day of CLP surgery for seven consecutive days. A total of 2 μL DASA‐58 (0.5 mg/mL) was infused into the lateral ventricle at a rate of 0.2 μL/min for 10 min using a syringe pump. The stereotaxic coordinates relative to bregma were AP: −0.50 mm; ML: +1.00 mm; and DV: −1.50 mm. The injection needle was left in place for an additional 5 min to prevent backflow.

### 2.4 Open Field Test, Contextual Fear Conditioning Test, and Y‐Maze Test

The open field test (OFT) was performed on Day 14 post‐CLP surgery, and the contextual fear conditioning test (CFCT) and Y‐maze test were performed on postoperative Day 15. The OFT, Y‐maze test, and CFCT were performed in accordance with previously published literature [24].

### 2.5 Golgi Staining and Nissl Staining

Fourteen days after CLP surgery, brain tissues were collected and subjected to Golgi staining to assess the dendritic spine density. The mice were randomly allocated to six groups. Two hippocampal CA1 pyramidal neurones were randomly selected from each mouse, and two intact second‐order apical dendritic segments were analyzed for each neuron. Briefly, mice were euthanised on Day 14 after CLP surgery, and brain tissues were immediately harvested and immersed in 10 mL of Golgi fixative solution. The tissues were fixed for 3 days at room temperature. The Golgi fixative was decanted. The tissue blocks were immersed in Golgi staining solution for 14 days, and the staining solution was renewed at 48 h and every 3 days thereafter. Subsequently, the brain tissues were sectioned at 150 μm with a vibrating microtome. The slides were rinsed with pure water for 5 min and incubated with concentrated ammonia water for 10 min. Finally, serial Z‐stack images were captured using confocal microscopy under identical settings, and spine density was quantified in a blinded manner.

The Nissl staining procedure was performed as previously described [24].

### 2.6 Transmission Electron Microscope

The preparation procedure for transmission electron microscopy (TEM) was performed as previously described [24]. For synapse quantification, complete synapses were identified using three standard ultrastructural features: presynaptic vesicle clusters, distinct synaptic cleft, and continuous postsynaptic density. To calculate synaptic density, we counted intact synapses in randomly selected equal‐area, non‐overlapping visual fields. For postsynaptic density (PSD) thickness measurements, for each intact PSD, a straight line perpendicular to the synaptic cleft was drawn across the PSD plaque. The linear distance from the inner edge of the synaptic cleft to the outermost cytoplasmic border of the PSD was measured using ImageJ software. All the quantitative analyses were performed in a blinded manner.

### 2.7 Glucose Assay

The hippocampal glucose content in mice was measured 24 h after CLP surgery. Briefly, the hippocampal tissues were isolated and processed into homogenates on ice at a 1:10 (w/v) ratio in distilled water. The homogenate was centrifuged at 12000 rpm for 10 min and the supernatant was harvested for subsequent detection. The microplate reader was set to a detection wavelength of 340 nm. According to the manufacturer's instructions for the Glucose Assay Kit (ADS‐W‐TDX040, AIDISHENG), 10 μL tissue supernatant was loaded into a 96‐well plate. The absorbance was read at 340 nm at 5 and 20 min post‐reaction, and the glucose concentration was determined using the standard formula provided.

### 2.8 ATP/ADP and NAD ^+^/NADH Ratio Assay

The ATP/ADP and NAD^+^/NADH ratios were measured in mice on Days 1 (two experimental groups) and 7 (four experimental groups) post‐CLP. Hippocampal ADP and ATP concentrations were quantified using an ADP/ATP Ratio Assay Kit (A552, DOJINDO) according to the manufacturer's protocol. Briefly, treated hippocampal tissues were lysed with an assay buffer. Baseline ATP luminescence was measured using a microplate luminometer, after which ADP in the samples was enzymatically converted into ATP. The total luminescence (ATP + ADP) was then determined, and the ATP/ADP ratio was calculated based on these luminescence values.

The concentrations of NAD^+^ and NADH in mouse hippocampal tissues were measured using the WST‐8 colorimetric assay (S0176S, Beyotime Biotechnology), followed by determination of the NAD^+^/NADH ratio. First, hippocampal tissues were lysed on ice and centrifuged at high speed at 4°C. The supernatant was collected for subsequent analysis. A 20 μL aliquot of the sample was combined with 90 μL alcohol dehydrogenase solution, incubated for 10 min at 37°C, followed by the addition of 10 μL chromogenic reagent to begin the reaction. The absorbance was read at 450 nm, and total NAD (NAD^+^ + NADH) was quantified using an NADH standard curve. Second, to degrade NAD^+^ and isolate NADH, samples were subjected to a water bath at 60°C for 30 min. The NAD^+^ concentration was determined by subtracting the measured NADH content from the total NAD content, which was then used to calculate the NAD^+^/NADH ratio. Protein concentrations in the hippocampal supernatant were assessed using the BCA method and all metabolite concentrations were normalized per unit of protein.

### 2.9 Primary Culture of Hippocampal Neurons

Hippocampal tissues were rapidly dissected from postnatal Day 0–1 (P0–P1) C57BL/6J mouse pups and placed in chilled Hibernate‐A medium. Hippocampal tissues were subjected to enzymatic digestion in a solution containing 0.05% trypsin and 0.05% DNase I at 37°C for 20 min in a water bath with gentle shaking. The tissues were washed three times with Neurobasal A medium (12,349,015, Thermo Fisher Scientific) supplemented with 1% penicillin–streptomycin and 10% heat‐inactivated foetal bovine serum and gently triturated using a truncated 1‐mL pipette tip, taking care to avoid bubble formation. After allowing the undigested tissue fragments to settle for 2 min, the supernatant was carefully collected and centrifuged at 100 × g for 5 min at room temperature. The cell pellet was collected and gently resuspended in complete culture medium consisting of Neurobasal A medium supplemented with 1% l‐glutamine, 2% B‐27 supplement, and 1% penicillin–streptomycin. Cells were then seeded into poly‐D‐lysine‐coated 6‐well plates at a density of 5 × 10^5^ cells/well in 2 mL of complete medium per well, which was designated as days in vitro 0 (DIV 0). Cells were maintained in a humidified incubator at 37°C with 5% CO2. To obtain relatively pure neuronal cultures, cytosine arabinoside (C1768, Sigma‐Aldrich) was added to the culture medium at a final concentration of 4 μM on DIV 3 to inhibit glial cell proliferation. On DIV 5, the medium was replaced with a fresh complete medium without Ara‐C. The medium was replaced every 3 days.

### 2.10 Cycloheximide (CHX) Chase Assay

CHX (C7698, Sigma‐Aldrich) was added to the culture medium of three groups of primary hippocampal neurones (LV‐Control + DMSO, LV‐Sec62 + DMSO, and LV‐Sec62 + BafA1) at a final concentration of 50 μg/mL. Total protein was extracted at 0, 3, 6, 9, and 12 h after CHX addition, with the 0 h time point collected immediately prior to CHX addition to define the baseline expression. Protein samples were analyzed by western blotting.

### 2.11 Primary Hippocampal Neurons (PHNs) With Treatment

On DIV 7, the PHNs were transduced with LV‐Sec62 or LV‐Control at a multiplicity of infection (MOI) of 10. Ten hours post‐transduction, the medium was completely replaced with fresh pre‐warmed complete medium, and subsequent medium changes were performed every 3 days. At DIV 11, overexpression of Sec62 was confirmed by western blotting in parallel cultures. On DIV 12, BafA1 was added to the designated group (LV‐Sec62) at a final concentration of 100 nM, whereas the control groups (LV‐Control and LV‐Sec62) received equal volumes of DMSO. After 2 h of pretreatment, the efficacy of BafA1‐mediated lysosomal inhibition was verified by western blotting in parallel cultures, as indicated by an increased LC3‐II/I amount and p62 accumulation. Subsequently, CHX was added to the three groups (LV‐Control + DMSO, LV‐Sec62 + DMSO, and LV‐Sec62 + BafA1).

### 2.12 Immunofluorescence

Brain tissues were collected from sham and SAE mice on postoperative Day 1. Then brain slices were incubated overnight at 4°C with the primary antibodies: phosphorylated STING (p‐STING, rabbit monoclonal, 1:100, ABclonal), Sec62 (rabbit monoclonal, 1:100; ABclonal), neuronal antibody (mouse monoclonal, 1:200, Abcam), and IBA1 (goat polyclonal, 1:200, Abcam). Then slices were incubated with secondary antibody buffer containing either Alexa Fluor 594 (donkey‐anti‐rabbit, 1:300, Invitrogen) or Alexa Fluor 488 (donkey‐anti‐goat or donkey‐anti‐mouse, 1:300, Invitrogen), followed by incubation for 2 h at 37°C in the dark. Following thorough washing, nuclei were stained with DAPI (1:1000, Abcam) for 10 min at room temperature. The sections were washed, cover‐slipped, air‐dried, and imaged using a confocal microscope (FV1200; Olympus, Japan).

### 2.13 Western Blot Analysis

Western blot (WB) analyses were conducted on mouse samples at 6 h and on Days 1, 2, 3, 7, and 14 post‐CLP in the two‐group experiment, and on Day 7 postoperative in the four‐group experiment. Protein samples were prepared, separated by electrophoresis, and transferred onto a polyvinylidene fluoride (PVDF) membrane. The membranes were incubated with the following primary antibodies: Sec62 (1:1000; ABclonal), p‐STING (1:500; ABclonal), STING (1:1000; ABclonal), postsynaptic density protein 95 (PSD95, 1:1000; ABclonal), PKM2 (1:1000; ABclonal), synapsin 1 (1:1000; ABclonal), and GAPDH (1:2000; ABclonal). After a 2 h incubation with the secondary antibody, chemiluminescent WB bands were visualized, captured, and densitometrically quantified using Image Lab software (Bio‐Rad, USA).

### 2.14 Statistical Analyses

GraphPad Prism software (version 9.0) was used for data analysis. Data are expressed as the mean ± standard deviation. The Shapiro–Wilk test was used to assess the normality of the data distribution. Group variances were assessed for homogeneity using the Brown‐Forsythe test for three or more groups and the F‐test for two groups. Statistical differences between groups were evaluated using one‐ or two‐way analysis of variance (ANOVA), followed by Tukey's post hoc test with Bonferroni correction to reduce false positives. An independent sample *t*‐test was performed to compare the mean values between the two groups. Golgi staining data were compared using nested *t*‐test or nested one‐way ANOVA. *p* < 0.05 was considered statistical significance.

## 3 Results

### 3.1 SAE Alters Glycolysis, Synaptic Plasticity and Behavioral Impairment

The results showed that the 14‐day survival rate was 50% (8/16) in the CLP mice (Figure S1B). While both groups showed similar motor function (Figure 1A–C, Figure S1C), behavioral assessments indicated that sepsis caused cognitive impairment (Figure 1A,D–I, Figure S1D,E). SAE mice showed a significant reduction in the number of hippocampal CA1 Nissl bodies at 7 and 14 days (Figure 1J,K) and significantly impaired synaptic plasticity at 14 days (Figure 1L–Q). On Day 1 following CLP‐induced sepsis, mice experienced significant decreases in hippocampal glucose concentrations, ATP/ADP NAD^+^/NADH ratios (Figure 1R–T). Concurrently, there was a significant reduction in synapsin 1 and PSD95 protein expression in the hippocampus (Figure 1U–W).

### 3.2 Enhancing PKM2 Activity Improves Glycolysis, Synaptic Plasticity and Behavioral Impairment in SAE Mice

In the hippocampal tissues of SAE mice, PKM2 expression and the tetramer‐to‐dimer ratio of PKM2 proteins significantly increased on Days 1 and 2 (Figure 2A–C, Figure S2A,B). The PKM2 agonist, DASA‐58, was used to assess the role of PKM2 in SAE mice (Figure 2A, Figure S2C,D). Our study revealed no differences in motor function among the different treatment groups (Figure 2D,E). However, the PKM2 agonist appeared to improve cognitive deficits in SAE mice (Figure 2F–K). Treatment with DASA‐58 significantly improved hippocampal CA1 synaptic plasticity in SAE mice (Figure 2L–Q). By Day 7, the treatment also induced a marked increase in hippocampal ATP/ADP and NAD^+^/NADH ratios, PKM2 concentrations, the PKM2 tetramer/dimer ratio, synapsin 1, and PSD95 compared to controls (Figure 2R–W, S2E,F).

### 3.3 P‐STING Expression Is Increased in the Hippocampal Tissue of SAE Mice

To characterize the dynamic changes in STING in the hippocampus of SAE mice, brain tissues were harvested 6 h, 1 day, 2 days, 3 days, 7 days, and 14 days after CLP surgery. Quantitative WB analysis showed that the expression levels of p‐STING/STING protein significantly increased on Days 1 and 2 post‐CLP (Figure 3A,B). In addition, we selected hippocampal tissues from SAE mice on Day 1 post‐CLP surgery for IF staining. The results indicated that p‐STING extensively colocalised with neurones in the hippocampal CA1 region of SAE mice (Figure 3C,D). Likewise, robust colocalisation between p‐STING and the microglial marker Iba1 was also detected within the hippocampal CA1 region (Figure 3E,F), suggesting that STING hyperactivation occurs in both neurones and microglia during the progression of SAE in mice.

**FIGURE 3:**
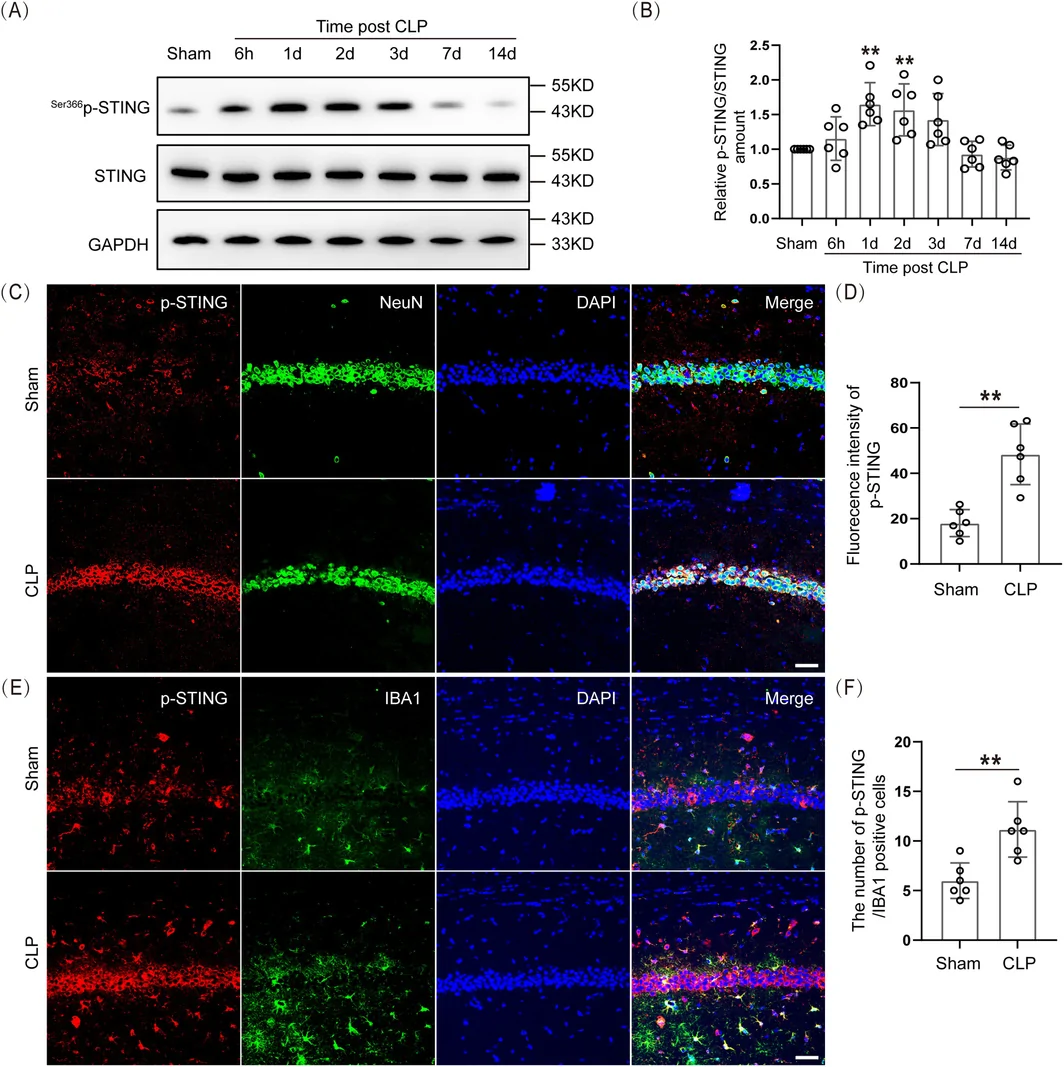
p‐STING expression is increased in the hippocampal tissue of SAE mice. (A) WB, (B) Statistical results of p‐STING/STING (*F* = 7.758, *p <* 0.0001). GAPDH was used as internal control. Statistical analysis: One‐way ANOVA with Dunnett's multiple comparisons test. (C, E) IF staining of p‐STING, scale bar 40 μm. (D, F) p‐STING statistical results. Statistical analysis: Student's *t*‐test. *p < 0.05, **p < 0.01 versus sham.

### 3.4 Knocking Down STING Improves Behavioral and Synaptic Plasticity Impairment via Enhancing PKM2 Activity in SAE Mice

To verify the effects of STING on cognitive function, synaptic plasticity, and PKM2 expression in SAE mice, STING expression was knocked down in the hippocampal CA1 area of 6‐week‐old mice using STING‐shRNA (Figure 4A, Figure S3A,B). No significant differences in motor ability were detected among the four groups of mice (Figure 4A–C); however, cognitive impairment significantly improved in SAE + STING‐shRNA mice (Figure 4A,D–I). On Day 7 post‐modeling, the hippocampal tissues of SAE + STING‐shRNA mice exhibited marked upregulation in synaptic plasticity (including synapsin 1 and PSD95 expression) alongside notable glycolysis enhancements, including increased ATP/ADP and NAD^+^/NADH ratios, higher total PKM2, and an increased PKM2 tetramer/dimer ratio (Figure 4J–R,U–W, Figure S3C,D). On Day 1 post modeling, STING‐shRNA effectively downregulated STING expression (Figure 4R–T). Our patch‐clamp recordings revealed that neuronal excitability in the hippocampal CA1 area markedly increased post‐CLP, whereas downregulation of STING expression reversed this phenotype (Figure S3E–G). Meanwhile, long‐term potentiation (LTP) was significantly impaired in the hippocampal CA1 area of SAE + scRNA mice; suppression of STING effectively rescued the CLP‐induced LTP deficits (Figure S3H–J). In addition, the mean amplitude of the miniature excitatory postsynaptic currents (mEPSCs) was not significantly different among the four experimental groups (Figure S3K,L). However, the mean frequency of mEPSCs markedly decreased in the SAE + scRNA group, and this reduction was fully restored by STING downregulation (Figure S3K,M).

**FIGURE 4:**
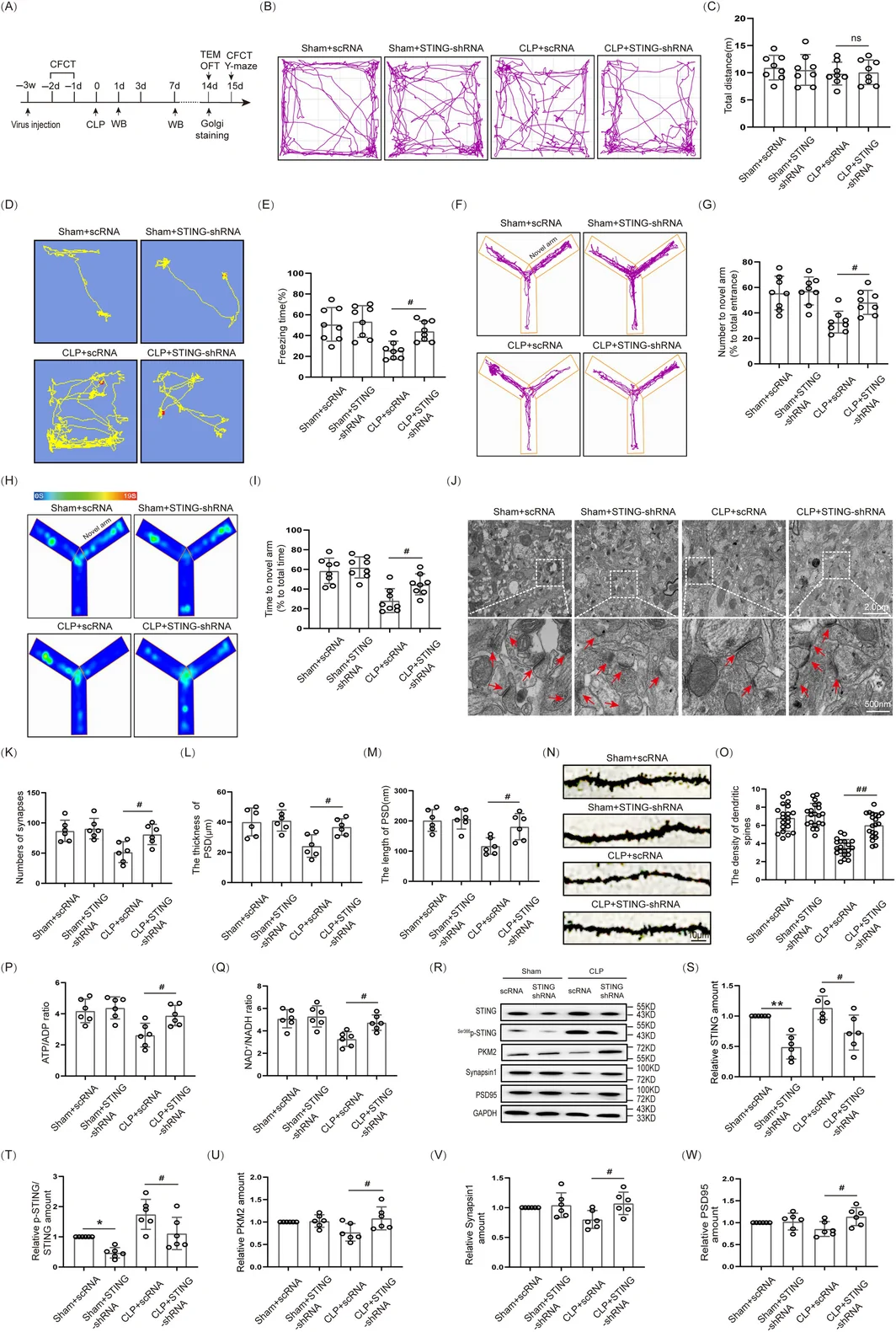
Knocking down STING improves behavioral and synaptic plasticity impairment via enhancing PKM2 activity in SAE mice. (A) Time diagram. (B) OFT trajectory diagram, (C) Total distance statistical results (*F* = 0.1568, *p* = 0.6951). (D) CFCT trajectory diagram, (E) Freezing time statistical results (*F* = 5.344, *p* = 0.0284). (F) Y‐maze test trajectory diagram, (G) Number to novel arm statistical results (*F* = 5.121, *p* = 0.0316). (H) Y‐maze test heatmap, (I) Time to novel arm statistical results (*F* = 5.876, *p* = 0.0221). (J) TEM images of synapses, (K–M) Synaptic plasticity statistical results (*F* = 4.967, *p* = 0.0375; *F* = 5.072, *p* = 0.0357; *F* = 5.667, *p* = 0.0273), scale bar 2.0 μm and 500 nm. Statistical analysis: Two‐way ANOVA with Sidak's multiple comparisons test. (N) Golgi staining, (O) Dendritic spine density statistical results (*F* = 24.79, *p* = 0.0002), scale bar 10 μm. Statistical analysis: Nested one‐way ANOVA with Dunnett's multiple comparisons test. (P, Q) Statistical results of ATP/ADP ratio (*F* = 6.057, *p* = 0.0231), NAD^+^/NADH ratio (*F* = 6.308, *p* = 0.0207). (R) WB, (S–W) Statistical results of STING (*F* = 31.34, *p* < 0.0001), p‐STING/STING (*F* = 14.35, *p* = 0.0012), PKM2 (*F* = 5.508, *p* = 0.0293), synapsin 1 (*F* = 5.752, *p* = 0.0263), and PSD95 (*F* = 5.311, *p* = 0.0320) protein expression detected by WB. GAPDH was used as internal control. Statistical analysis: Two‐way ANOVA with Sidak's multiple comparisons test. *p < 0.05, **p < 0.01 versus Sham + scRNA; ns: No significant, *p* > 0.05, ^#^ *p* < 0.05, ^##^ *p* < 0.01 versus CLP + scRNA.

To investigate the functional effects of STING on microglia, we injected a microglia‐specific STING knockdown virus into the hippocampal CA1 area of 6‐week‐old mice (Figure 4A). After 3 weeks, sepsis was induced by CLP. The microglia‐specific STING knockdown virus effectively downregulated STING expression in the hippocampal tissue (Figure S4A–C). Behavioral results revealed no significant differences in motor activity among the four groups of mice (Figure S4D,E). Strikingly, cognitive impairment significantly improved in SAE + hIBA1a‐STING‐shRNA mice (Figure S4F–K).

### 3.5 STING Downregulation Does Not Improve Behavioral Impairment in PKM2 cKO SAE Mice

To further demonstrate the functional role of STING in the regulation of PKM2 expression, PKM2 cKO mice were used in this study. The OFT results indicated no differences in motor function among the different groups of mice (Figure 5A–C,J,K; Figure S5A,B). PKM2 cKO mice exhibited significant cognitive impairment compared to their control littermates (Figure S5C–H). In addition, no significant difference in cognitive performance was observed between SAE + Control and SAE + PKM2 cKO mice (Figure 5D–I). Second, we injected STING‐knockdown virus into the hippocampal CA1 region of control and PKM2 cKO mice (Figure 5A). Our results revealed that cognitive impairment was not ameliorated in STING‐shRNA + SAE + PKM2 cKO mice compared to that in STING‐shRNA + SAE + Control mice (Figure 5L–Q). We performed PCR genotyping and western blot analysis to verify the successful construction of neuron‐specific PKM2 cKO mice (Figure S5I, Figure 5R,S).

**FIGURE 5:**
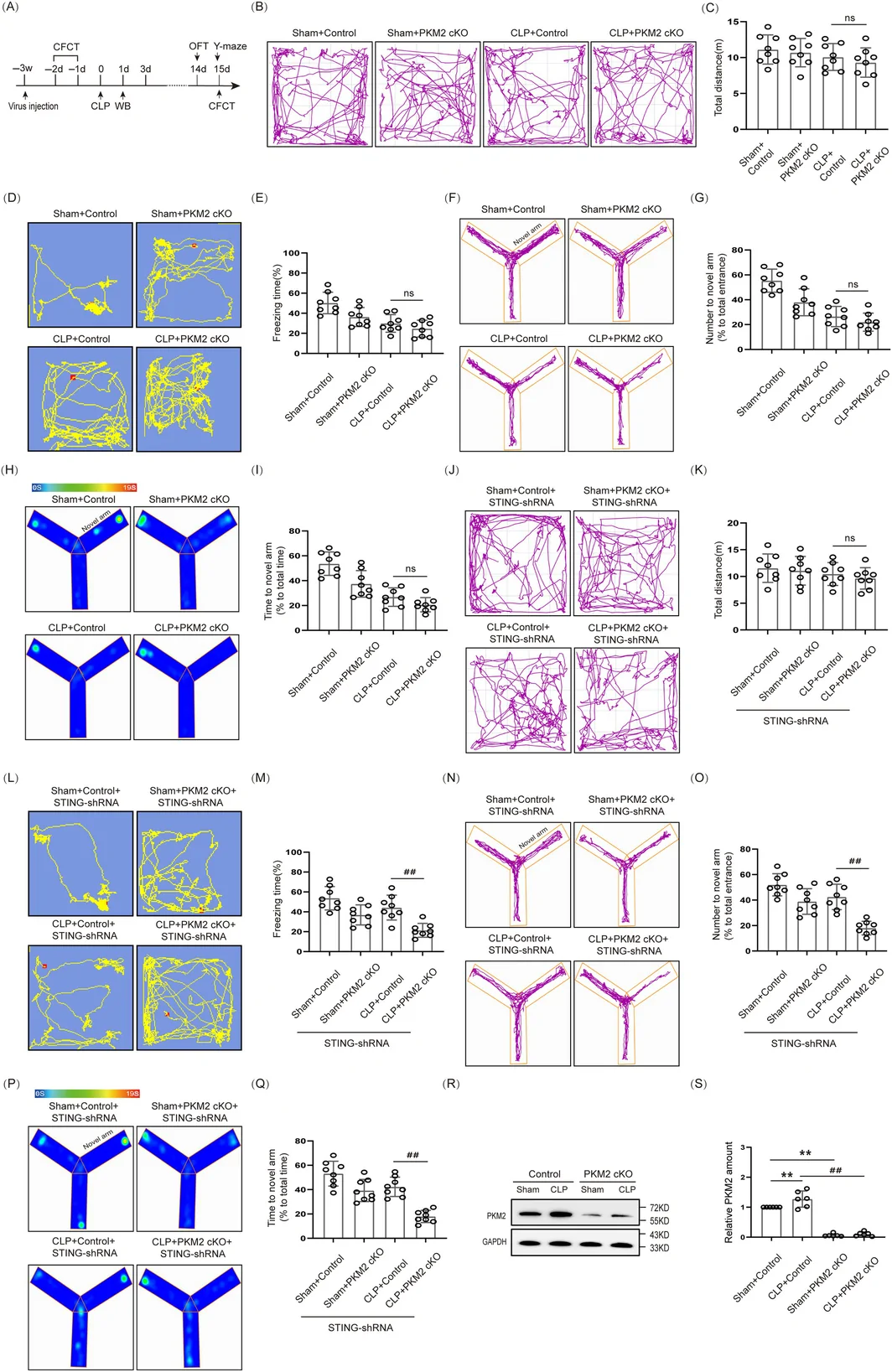
STING downregulation does not improve behavioral impairment in PKM2 cKO SAE mice. (A) Time diagram. (B, J) OFT trajectory diagram, (C, K) Total distance statistical results (*F* = 0.05676, *p* = 0.8134; *F* = 0.03884, *p* = 0.8452). (D, L) CFCT trajectory diagram, (E, M) Freezing time statistical results (*F* = 1.784, *p* = 0.1924; *F* = 25.04, *p* < 0.0001). (F, N) Y‐maze test trajectory diagram, (G, O) Number to novel arm statistical results (*F* = 4.124, *p* = 0.052; *F* = 37.25, *p* < 0.0001). (H, P) Y‐maze test heatmap, (I, Q) Time to novel arm statistical results (*F* = 2.765, *p* = 0.1075; *F* = 42.58, *p* < 0.0001). (R) WB, (S) Statistical results of PKM2 (*F* = 409.5, *p* < 0.0001). GAPDH was used as internal control. Statistical analysis: Two‐way ANOVA with Sidak's multiple comparisons test. ns: No significant, *p* > 0.05 versus CLP + Control or STING‐shRNA + CLP + Control, **p < 0.01 versus Sham + Control, ^##^ *p* < 0.01 versus CLP + Control or STING‐shRNA + CLP + Control.

### 3.6 Enhanced Micro‐ER‐Phagy Alleviates Behavioral Impairment and Improves Synaptic Plasticity Deficits in SAE Mice by Reversing STING‐Associated Inhibition of PKM2 Activity

At 1, 2, and 3 days post‐CLP, a significant increase in Sec62 and LC3 II/LC3 I protein expression levels was noticed in the hippocampal tissue of the mice (Figure 6A–C and Figure S5J,K). p62 protein expression levels in the hippocampal tissue of mice were markedly decreased 6 h after CLP (Figure S5J,L). Sec62 protein and lysosomes were significantly upregulated in the neurones of the hippocampal CA1 area in SAE mice (Figure S5M–O).

**FIGURE 6:**
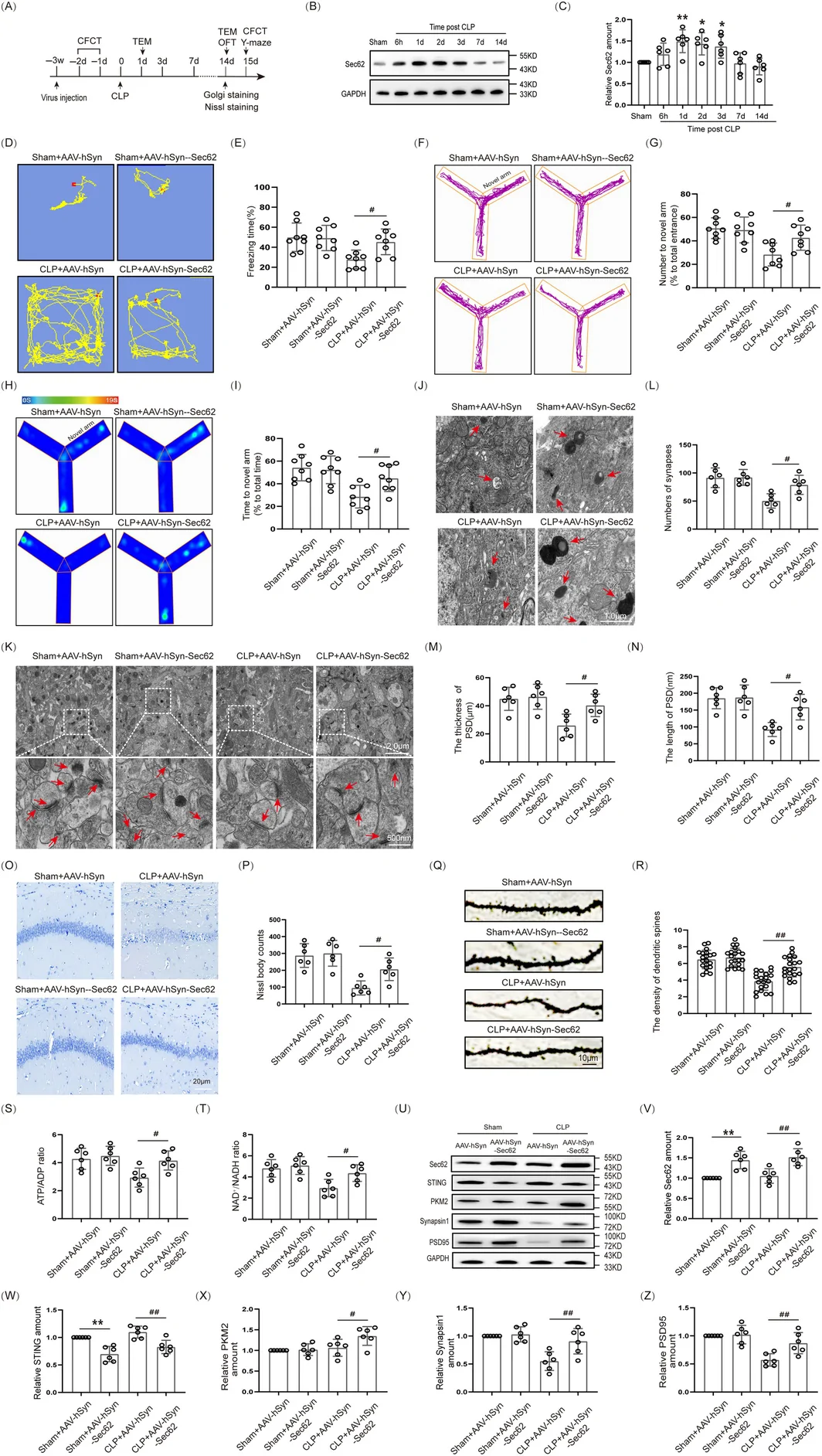
Enhanced micro‐ER‐phagy alleviates behavioral impairment and improves synaptic plasticity deficits in SAE mice by reversing STING‐associated inhibition of PKM2 activity. (A) Time diagram. (B, U) WB, (C) statistical results of Sec62 (*F* = 6.388, *p* = 0.0001). GAPDH was used as internal control. Statistical analysis: One‐way ANOVA with Dunnett's multiple comparisons test. *p < 0.05, **p < 0.01 versus sham. (D) CFCT trajectory diagram, (E) Freezing time statistical results (*F* = 8.815, *p* = 0.0061), (F) Y‐maze test trajectory diagram, (G) Number to novel arm statistical results (*F* = 4.897, *p* = 0.0352). (H) Y‐maze test heatmap, (I) Time to novel arm statistical results (*F* = 5.047, *p* = 0.0327). Statistical analysis: Two‐way ANOVA with Sidak's multiple comparisons test. (J) TEM images of lysosomes, scale bar 1.0 μm. (K) TEM images of synapses, (L–N) Synaptic plasticity statistical results (*F* = 4.847, *p* = 0.0396; *F* = 5.400, *p* = 0.0308; *F* = 5.856, *p* = 0.0252), scale bar 2.0 μm and 500 nm. Statistical analysis: Two‐way ANOVA with Sidak's multiple comparisons test. (O) Nissl staining, (P) Nissl bodies count statistical results (*F* = 5.426, *p* = 0.0304), scale bar 20 μm. (Q) Golgi staining, (R) Dendritic spine density statistical results (*F* = 21.69, *p* = 0.0020), scale bar 10 μm. Statistical analysis: Nested one‐way ANOVA with Dunnett's multiple comparisons test. (S, T, V–Z) Statistical results of ATP/ADP ratio (*F* = 6.758, *p* = 0.0171) and NAD^+^/NADH ratio (*F* = 6.300, *p* = 0.0208), Sec62 (*F* = 40.95, *p* < 0.001), STING (*F* = 43.27, *p* < 0.001), PKM2 (*F* = 4.9930, *p* = 0.0370), synapsin 1 (*F* = 9.082, *p* = 0.0069), PSD95 (*F* = 19.12, *p* = 0.0003). GAPDH was used as internal control. Statistical analysis: Two‐way ANOVA with Sidak's multiple comparisons test. **p < 0.01 versus Sham + AAV‐hSyn; ^#^ *p* < 0.05, ^##^ *p* < 0.01 versus CLP + AAV‐hSyn.

To verify the role of micro‐ER‐phagy in SAE mice, we injected the Sec62 overexpression virus into the hippocampal CA1 area of 6‐week‐old mice (Figure 6A, Figure S5P,Q). There was no significant difference in the motor activity between the experimental groups (Figure S5R,S). Interestingly, enhancing micro‐ER‐phagy ameliorated cognitive impairment in SAE mice (Figure 6D–I). Lysosomes, synaptic plasticity, and neurones in the hippocampal CA1 area were significantly elevated in SAE + AAV‐hSyn‐Sec62 mice (Figure 6J–R). In the hippocampi of SAE + AAV‐hSyn‐Sec62 mice, a significant increase was observed in the ATP/ADP ratio, NAD^+^/NADH ratio, Sec62, total PKM2, PKM2 tetramer/dimer ratio, synapsin 1, and PSD95 concentrations (Figure 6S–V,X–Z and Figure S5T,U).

STING is degraded via the lysosomal pathway. First, we performed qRT‐PCR on the hippocampal tissues from Sham+AAV‐hSyn, SAE + AAV‐hSyn, and SAE + AAV‐hSyn‐Sec62 mice. Sec62 mRNA was significantly upregulated in the SAE + AAV‐hSyn‐Sec62 group compared to the SAE + AAV‐hSyn control group, confirming the successful overexpression of Sec62 in the hippocampus (Figure S6A). However, the STING mRNA expression levels remained unchanged in the SAE + AAV‐hSyn‐Sec62 and SAE + AAV‐hSyn groups (Figure S6B). Next, we performed a cycloheximide (CHX) chase assay with Bafilomycin A1 (BafA1) to block lysosomal degradation. We first verified the effectiveness of LV‐Sec62 virus and BafA1‐mediated lysosomal inhibition in PHNs via WB (Figure S6C–G). Subsequently, we found that Sec62 overexpression significantly shortened the half‐life of STING compared to that in vector‐transduced cells, whereas co‐treatment with BafA1 effectively reversed this effect (Figure S6H,I). In addition, the upregulation of Sec62 expression enhanced autophagy and decreased STING expression (Figure S6J–L, Figure 6U–W). However, we treated mice with BafA1 to inhibit lysosomal activity and found that the expression levels of Sec62, LC3 II/LC3 I, p62, and STING proteins in the hippocampi of SAE + BafA1 mice were significantly increased (Figure S6M–Q). Collectively, these findings demonstrate that Sec62 promotes STING degradation at the post‐translational level through the autophagy‐lysosomal pathway rather than by modulating STING transcription or translation.

## 4 Discussion

Survivors of SAE frequently develop long‐term cognitive impairment [2, 3], which was confirmed in our SAE mouse model using the Y‐maze test and CFCT [30, 31, 32]. This cognitive decline is primarily driven by hippocampal neuronal death [33, 34], specifically in the CA1 area, which plays a crucial role in learning and memory [14, 35]. Our results further revealed neuronal loss and synaptic plasticity impairments in the hippocampal CA1 region of SAE mice. Further investigation revealed insufficient energy production from glycolysis in the hippocampal tissues of SAE mice.

Synaptic activity requires large amounts of ATP to provide energy [6, 36, 37, 38, 39]. Under various forms of stress, PKM2 drives glycolysis to meet this demand [15, 16, 17, 18]. Consequently, PKM2 inactivation can lead to energy deficits and impaired synaptic plasticity [40, 41]. Our results showed an increase in PKM2 expression and a higher PKM2 tetramer‐to‐dimer ratio in the hippocampi of SAE mice. We hypothesized that this upregulation was a stress‐induced adaptive response. However, this increase was too modest to provide neuroprotection, ultimately failing to prevent cognitive impairment in SAE mice. Surprisingly, enhanced PKM2 activity rescued synaptic plasticity and cognitive deficits in these mice, indicating that PKM2 plays a critical role in the pathophysiology of SAE.

STING activates the TANK‐binding kinase 1/nuclear factor kappa B pathway to control energy metabolism and inflammation [20, 21, 22, 42]. However, the mechanism by which STING regulates glycolysis in the hippocampi of SAE mice remains unclear. Our results show that p‐STING/STING expression was significantly elevated in the hippocampi of SAE mice. Additionally, we unexpectedly observed that the knockdown of STING expression in neurones upregulated PKM2 activity. This metabolic shift supplies additional energy to support synaptic plasticity, ultimately alleviating cognitive impairment in SAE mice. To further explore this observation, we investigated whether STING regulates synapses through PKM2, thereby affecting cognitive function in SAE mice. Surprisingly, STING knockdown did not rescue the cognitive deficits observed in SAE PKM2 cKO mice. Given that PKM2 cKO mice exhibited significant cognitive impairments compared to control littermates in the baseline behavioral characterization, we speculated that the loss of the protective effect of STING knockdown in PKM2 cKO mice may reflect not only PKM2‐dependent pathway specificity but also pre‐existing dysfunction caused by PKM2 deletion, consistent with previous reports that PKM2 loss impairs cognition and alters neuronal metabolism [16, 23]. Therefore, we acknowledge this as a limitation of the present study. Based on these findings, we propose that STING‐associated inhibition of PKM2 activity in the hippocampus impairs synaptic plasticity, thereby contributing to cognitive dysfunction in SAE mice. In addition, our results show that reducing STING expression in microglia ameliorates cognitive dysfunction in SAE mice. However, specific regulatory mechanisms in microglia remain to be elucidated in our future study.

Studies have indicated that SEC62 is critical for micro‐ER‐phagy and ER recovery [25, 26, 43]. Because SEC62 and STING share a similar LC3‐interacting region domain [26, 27, 28], we hypothesized that upregulating micro‐ER‐phagy activity would promote STING degradation. Our results showed that SEC62 overexpression enhanced autophagy and accelerated STING degradation, whereas autophagy inhibition blocked these effects, which is consistent with a previous report [44]. Furthermore, our results demonstrated that the enhancement of micro‐ER‐phagy activity improved cognitive impairment, alleviated synaptic dysfunction, and increased PKM2 activity in SAE mice. Collectively, these findings suggest that enhancing micro‐ER‐phagy promotes the autophagic degradation of STING, thereby relieving the inhibition of PKM2 activity, increasing energy supply, reducing synaptic damage, and ultimately improving cognitive function in SAE mice.

Our study had several limitations. Firstly, we did not test whether DASA‐58‐mediated protection is abolished in PKM2 cKO mice; thus, the strict PKM2‐dependency of DASA‐58 remains incompletely established. This limitation needs to be addressed in future studies. Second, direct morphological evidence for lysosomal trafficking of STING will be pursued in future studies.

## 5 Conclusion

In summary, the STING‐mediated inhibition of PKM2 activity triggered defective synaptic plasticity and cognitive impairment in SAE mice, whereas boosting micro‐ER‐phagy reversed these pathological phenotypes. This conclusion may provide a promising mechanistic direction for the clinical treatment of SAEs.

## Author Contributions

Xiaofeng Guo, Daqing Yin and Zhonghua Zhang wrote the manuscript. Yuanyuan Zhao, Yali Zhang, Ying Jiang and Zhanying Zhao analyzed the data and performed the experiments. Tongwen Sun, Feihu Zhou and Xijing Zhang revised the manuscript.

## Funding

This work was supported by the National Natural Science Foundation of China, 82272190 and Natural Science Foundation of Henan Province, 252300421400.

## Ethics Statement

This study was approved by the committee on ethics of medicine of the 988th hospital of joint logistics support forces with approval number (Grant No. PLA98820240919LLSP).

## Conflicts of Interest

The authors declare no conflicts of interest.

## Supporting information


**Figure S1:** The full‐length coding sequence (CDS) of mouse Sec62, survival rate and diagram of behavioral. (A) The full‐length CDS of mouse Sec62, (B) The 14‐day survival rate of the septic mice, (C) OFT diagram, (D) CFCT diagram, (E) Y‐maze test diagram.

**Figure S2:** PKM2 oligomer in SAE mouse hippocampus and the experimental schematic of intracerebroventricular injection of PKM2 agonist. (A, E) WB, (B) Statistical results of the PKM2 tetramer/dimer (*F* = 5.876, *p* = 0.0003). Tubulin was used as internal control. Statistical analysis: one‐way ANOVA with Dunnett's multiple comparisons test. *p < 0.05, **p < 0.01 versus sham. (C) Diagram of PKM2 agonist injection, (D) Cerebroventricular cannulation diagram. (F) Statistical results of PKM2 tetramer/dimer) (*F* = 5.194, *p* = 0.0338). Tubulin was used as internal control. Statistical analysis: two‐way ANOVA with Sidak's multiple comparisons test. ^#^ *p* < 0.05 versus CLP + DMSO.

**Figure S3:** STING knockdown in hippocampal CA1 neurons elevates PKM2 oligomer and restores neuronal electrophysiological function in SAE mice. (A) Diagram of virus injection, (B) Downregulation of STING virus transfection map. (C) WB, (D) Statistical results of PKM2 tetramer/dimer (*F* = 5.839, *p* = 0.0254). Tubulin was used as internal control. Statistical analysis: two‐way ANOVA with Sidak's multiple comparisons test. (E) Typical traces of neuronal firing activity in response to intracellular injection +100 pA depolarizing current, (F) Mean RMP of CA1 pyramidal neurons (*F* = 4.564, *p* = 0.0415), (G) Number of spikes in response to different depolarizing currents in the four groups of CA1 pyramidal neurons (*F* = 148.6, *p* < 0.0001, Statistical analysis: two‐way repeated ANOVA with Bonferroni's post hoc test). (H) Schematic representation of fEPSPs before (1) and after (2) HFS among the four groups. Horizontal calibration bars, 10 ms; vertical bars, 0.5 mV. (I) Time‐dependent changes in the slope of fEPSPs before (1) and after (2) HFS in hippocampal slices among the four groups, (J) Quantification of fEPSP slopes evoked by HFS (*F* = 10.81, *p* = 0.0027). (K) Samples of sEPSCs recorded in CA1 pyramidal neurons, (L) Quantification of mean amplitude of mEPSCs (*F* = 0.1937, *p* = 0.6632), (M) Quantification of mean frequency of mEPSCs (*F* = 5.659, *p* = 0.0244). Statistical analysis: two‐way ANOVA with Sidak's multiple comparisons test. ns: no significant, *p* > 0.05, ^#^ *p* < 0.05, ^##^ *p* < 0.01 versus CLP + scRNA.

**Figure S4:** Microglia‐specific STING knockdown ameliorates behavioral impairment in SAE mice. (A) WB, (B, C) Statistical results of STING (*F* = 66.09, *p* < 0.0001), p‐STING/STING (*F* = 43.00, *p* < 0.0001) protein expression detected by WB. GAPDH was used as internal control. (D) OFT trajectory diagram, (E) Total distance statistical results (*F* = 0.0277, *p* = 0.8689). (F) CFCT trajectory diagram, (G) freezing time statistical results (*F* = 4.708, *p* = 0.0387). (H) Y‐maze test trajectory diagram, (I) Number to novel arm statistical results (*F* = 4.780, *p* = 0.0373). (J) Y‐maze test heatmap, (K) Time to novel arm statistical results (*F* = 5.901, *p* = 0.0218). Statistical analysis: two‐way ANOVA with Sidak's multiple comparisons test. **p < 0.01 versus Sham + hIBA1a‐scRNA; ns: no significant, *p* > 0.05, ^#^ *p* < 0.05, ^##^ *p* < 0.01 versus CLP + hIBA1a‐scRNA.

**Figure S5:** Behavioral profiles of PKM2 cKO mice, micro‐ER‐phagy detection, viral transfection map, PKM2 oligomer measurement. (A, R) OFT trajectory diagram, (B) Total distance statistical results. (C) CFCT trajectory diagram, (D) Freezing time statistical results. (E) Y‐maze test trajectory diagram, (F) Number to novel arm statistical results. (G) Y‐maze test heatmap, (H) Time to novel arm statistical results. (I) Typical PCR identification image of PKM2 cKO mice. (J, T) WB, (K, L) Statistical results of LC3 II/LC3 I (*F* = 18.93, *p* < 0.0001) and p62 (*F* = 8.262, *p* < 0.0001). GAPDH was used as internal control. Statistical analysis: One‐way ANOVA with Dunnett's multiple comparisons test. *p < 0.05, **p < 0.01 versus sham. (M) IF staining of Sec62, scale bar 40 μm, (N) statistical results of Sec62. Statistical analysis: Student's *t*‐test. **p < 0.01 versus sham. (O) TEM images of lysosomes, scale bar 1.0 μm. (P) Diagram of virus injection, (Q) Virus transfection map. (S) Total traveling distance statistical results (*F* = 0.1418, *p* = 0.7093), (U) statistical results of PKM2 (tetramer)/PKM2 (dimer) (*F* = 4.972, *p* = 0.0374). Tubulin was used as internal control. Statistical analysis: two‐way ANOVA with Sidak's multiple comparisons test. ns: no significant, *p* > 0.05, ^#^ *p* < 0.05 versus CLP + AAV‐hSyn.

**Figure S6:** STING is degraded through the lysosomal pathway. (A) Relative mRNA expression of Sec62 (*F* = 47.01, *p* < 0.0001). (B) Relative mRNA expression of STING (*F* = 8.318, *p* = 0.0037). Statistical analysis: one‐way ANOVA with Tukey's multiple comparisons test. ns: no significant, *p* > 0.05, **p < 0.01 versus CLP + AAV‐hSyn. (C, E, H, J, M) WB, (D, F, G) Statistical results of Sec62, LC3 II/LC3 I and p62. GAPDH was used as internal control. Statistical analysis: Student's *t*‐test. **p < 0.01 versus PHNs + LV‐Control or PHNs + LV‐Control + DMSO. (I) Statistical analysis of STING protein half‐life (*F* = 70.01, *p* < 0.0001). Statistical analysis: one‐way ANOVA with Tukey's multiple comparisons test, **p < 0.01 versus PHNs + LV‐Control + CHX, ^##^ *p* < 0.01 versus PHNs + LV‐Sec62 + CHX. (K, L) Statistical results of LC3 II/LC3 I (*F* = 29.38, *p* < 0.0001) and p62 (*F* = 37.23, *p* < 0.0001). GAPDH was used as internal control. Statistical analysis: two‐way ANOVA with Sidak's multiple comparisons test. **p < 0.01 versus Sham + AAV‐hSyn, ^##^ *p* < 0.01 versus CLP + AAV‐hSyn. (N‐Q) Statistical results of Sec62 (*F* = 20.65, *p* = 0.0002) and LC3 II/LC3 I (*F* = 19.44, *p* = 0.0003), p62 (*F* = 24.75, *p* < 0.0001), STING (*F* = 27.51, *p* < 0.0001). GAPDH was used as internal control. Statistical analysis: two‐way ANOVA with Sidak's multiple comparisons test. *p < 0.05 versus Sham + DMSO, ^#^ *p* < 0.05, ^##^ *p* < 0.01 versus CLP + DMSO.

## Data Availability

The data that support the findings of this study are available from the corresponding author upon reasonable request.
